# Efficacy of adjuvant chemotherapy/maintenance chemotherapy after induction chemotherapy and concurrent chemoradiotherapy in patients with locoregionally advanced nasopharyngeal carcinoma: Experiences of two centers

**DOI:** 10.1002/cam4.5470

**Published:** 2022-11-24

**Authors:** Hao‐Yun Tao, Fang He, Qi‐Yun Shi, Ran Liu, Zhi‐Long Wang, Kun‐Peng Du, Jian‐Feng Li, Hui Liu, Zhi‐Qiang Lu, Jing‐Jing Zhang, Yu‐Hai Bai

**Affiliations:** ^1^ Department of Radiation Oncology Guangzhou Medical University Cancer Center Guangzhou China; ^2^ Department of Radiation Oncology People's Hospital of Zhongshan City Zhongshan China; ^3^ Department of Breast and Thyroid Surgery, Southwest Hospital Army Medical University Chongqing China; ^4^ Department of Pediatrics LongHua District People's Hospital Shenzhen China; ^5^ Department of Radiology People's Hospital of Zhongshan City Zhongshan China

**Keywords:** adjuvant chemotherapy, induction chemotherapy, locoregionally advanced nasopharyngeal carcinoma, maintenance chemotherapy, nomogram, S‐1

## Abstract

**Background and Objective:**

In general, there are not many studies exploring the clinical value of adjuvant chemotherapy or maintenance chemotherapy (AC/MC) after induction chemotherapy and concurrent chemoradiotherapy (IC+CCRT+AC/MC). The purpose of this study was to establish a clinical nomogram for the use of AC/MC in patients with locoregionally advanced nasopharyngeal carcinoma (LA‐NPC).

**Material and Methods:**

Two centers (Guangzhou Medical University Cancer Center [*N* = 1226] and Zhongshan People's Hospital [*N* = 150]) recruited 1376 patients with LA‐NPC. All the patients underwent IC+CCRT; 560 patients received AC with cisplatin/nedaplatin plus docetaxel/paclitaxel (TP) or cisplatin/nedaplatin plus fluorouracil (PF), and 81 patients received MC with S‐1. Multivariate Cox regression was used to confirm optimal predictors of progression‐free survival (PFS), and a nomogram was established to identify patients into low‐risk and high‐risk cohorts. Additionally, bootstrap internal validation was performed to further verify our nomogram.

**Results:**

After propensity score matching (PSM), the survival curves were not statistically different between IC+CCRT+AC/MC and IC+CCRT (all *p* > 0.05). Then, a nomogram was developed based on variables that were screened by univariate and multivariate Cox regression, including N stage, cumulative platinum dose during CCRT, body mass index (BMI), IC cycles, IC regimen and cervical lymph node (CLN) necrosis and infiltration of adjacent tissues. The results of the nomogram showed that the high‐risk cohort had greatly worse 5‐year DMFS, LRFS, PFS and OS compared to low‐risk cohort (all *p* < 0.05), and subgroup analysis found that the 5‐year DMFS, PFS and OS of patients treated with IC+CCRT+AC/MC were better than those treated with IC+CCRT in high‐risk cohort (all *p* < 0.05). Notably, the incidence of adverse effects for IC+CCRT+AC cohort was higher than that for IC+CCRT+MC cohort, especially leukocytopenia and neutropenia. IC+CCRT and IC+CCRT+MC were associated with similar incidences of adverse effects.

**Conclusions:**

The addition of AC or MC to IC+CCRT could improve the DMFS of patients with high‐risk NPC and prolong their survival. Additionally, our findings suggest a potential role of AC/MC following IC plus CCRT in the treatment of high‐risk LA‐NPC.

## Graphical abstract

Efficacy of adjuvant chemotherapy/maintenance chemotherapy after induction chemotherapy and concurrent chemoradiatherapy in patients with locoregionally advanced nasopharyngeal carcinoma.

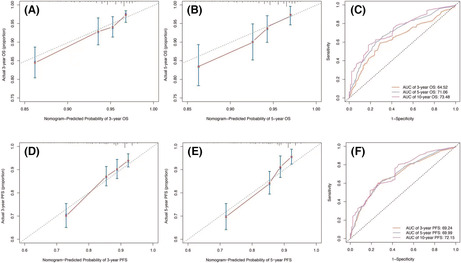

## 1 INTRODUCTION

The incidence of nasopharyngeal carcinoma (NPC) is associated with geographical distribution, and a considerable number of individuals in Southeast Asia and southern China are diagnosed with NPC each year.^1^ Due to the anatomical complexity and radiosensitivity of NPC, radiotherapy is the preferred treatment option.^2^ Local control rates improve significantly with intensity‐modulated radiation therapy (IMRT). Nevertheless, distant metastasis is the leading cause of treatment failure in patients with locoregionally advanced nasopharyngeal carcinoma (LA‐NPC).^3^

Stage II‐IVA NPCs are usually treated with concurrent chemoradiotherapy (CCRT), per National Comprehensive Cancer Network (NCCN) guidelines.^4^ In recent years, numerous prospective trials have shown that adding induction chemotherapy (IC) to CCRT can lead to longer survival and a lower incidence of distant metastasis in LA‐NPC patients.^5^, ^6^ Therefore, IC+CCRT has been upgraded to the 2A evidence category.^4^ However, approximately 30% of patients with LA‐NPC ultimately experience relapse or metastasis even after treatment with IC+CCRT.^5^, ^6^, ^7^, ^8^

According to Chen et al., maintenance chemotherapy (MC) with capecitabine can further increase the survival rate of LA‐NPC patients.^9^ Tao et al.'s results showed that patients with N2‐3 positivity could benefit from adjuvant chemotherapy (AC) following IC+CCRT.^10^ Additionally, studies have demonstrated that cervical lymph node (CLN) necrosis and infiltration of adjacent structures can adversely affect patient prognosis.^11^, ^12^, ^13^ In this article, we screened the essential prognostic factors based on imaging data and clinical characteristics to further explore the value of AC or MC following IC+CCRT in patients with LA‐NPC.

## 2 MATERIALS AND METHODS

### 2.1 Patients and follow‐up

We enrolled 1376 LA‐NPC patients who were diagnosed at the Zhongshan People's Hospital (Zhongshan = 150) from February 2014 to December 2017 and the Affiliated Cancer Hospital & Institute of Guangzhou Medical University from January 2010 to July 2017 (Guangzhou = 1226). The UICC/AJCC 8th edition staging system^14^, ^15^ was used to restage all patients who met all of the following criteria: (1) newly diagnosed with stage III–IVa NPC; (2) underwent magnetic resonance imaging (MRI) examination before starting treatment; (3) received IC with cisplatin/nedaplatin (DDP/NDP) plus docetaxel/paclitaxel (TP), DDP/NDP plus fluorouracil (PF), or docetaxel/paclitaxel and fluorouracil (TPF); (4) treated with CCRT using DDP/NDP; (5) treated with or without AC or MC; (6) available clinical data and blood and nonblood examination results; and (7) underwent definitive intensity‐modulated radiotherapy (IMRT). The ethics committees for Guangzhou Medical University's Affiliated Cancer Hospital and Zhongshan People's Hospital approved this research.

### 2.2 Treatment

All patients were immobilized in the supine position; their head, neck and shoulders were covered with a thermoplastic mask; and they were then scanned by using a computerized tomography simulator. Scanning was performed from the vertex of the skull to 2 cm below the head of the clavicle, with slices of 3 mm. The target volume was delineated based on noncontrast and contrast magnetic resonance imaging (MRI) and CT images, and it included the gross tumor volume of nasopharyngeal tumors (GTVnx), gross tumor volume of lymph nodes (GTVnd), high‐risk clinical target volume (CTV1) and low‐risk clinical target volume (CTV2). Sun Yat‐sen University Cancer Center institutional treatment protocols,^16^ in agreement with the International Commission on Radiation Units and Measurements Reports 50 and 62, were used to contour the target volumes. The radiation doses of GTVnx, GTVnd, CTV1 and CTV2 were 70–74 Gy, 68–70 Gy, 60–66 Gy and 54–56 Gy, respectively, with 30 to 32 fractions in total. All the patients received IC every 3 weeks for a maximum of 4 cycles; 346 patients received cisplatin/nedaplatin (65 mg/m^2^ d1), docetaxel (65 mg/m^2^ d1)/paclitaxel (135 mg/m^2^) and fluorouracil (600 mg/m^2^, 24 h a day for d1‐5) (TPF), 813 patients received cisplatin (DDP)/nedaplatin (NDP) (70–80 mg/m^2^) plus docetaxel (65–75 mg/m^2^ d1)/paclitaxel (135 mg/m^2^) (TP), and 217 patients received DDP/NDP (70–80 mg/m^2^ d1) plus fluorouracil (1000 mg/m^2^, 24 h a day for d1‐5) (PF). During CCRT, 1226 patients were given DDP/NDP (70–80 mg/m^2^ d1) every three weeks for 1–4 cycles at Guangzhou Medical University, and 150 patients were treated with 6 cycles of DDP/NDP (30–40 mg/m^2^, d1) every week at Zhongshan People's Hospital. After CCRT, 641 patients received AC (*n* = 560) or MC (*n* = 81). In the AC group, 409 patients received DDP/NDP (70–80 mg/m^2^ d1) plus docetaxel (65–75 mg/m^2^ d1)/paclitaxel (135 mg/m^2^), and 151 patients received DDP/NDP (70–80 mg/m^2^ d1) plus fluorouracil (1000 mg/m^2^, 24 h a day for d1‐5). Of 560 patients, 169 (30.2%) patients received one course of AC, and 391 (69.8%) patients received 2 to 4 courses of AC. In the MC group, 81 patients were given S‐1 (40–60 mg bid d1–d14) for at least two cycles. The dosage of S‐1 depended on the body surface area (BSA): patients with a BSA≤1.25 m^2^, 1.25 m^2^ < BSA <1.5 m^2^ or BSA≥1.5 m^2^ were given 40, 50, or 60 mg twice a day, respectively.

### 2.3 Imaging data

All the patients underwent pretreatment MRI scanning from the vertex of the skull to 2 cm below the clavicular head using a 1.5‐ or 3.0‐T system (Philips Medical Systems, Best, the Netherlands), with slices of 3 or 1.5 mm. Two radiotherapy doctors at Guangzhou Medical Cancer Center and a radiotherapy doctor and a radiologist at Zhongshan People's Hospital used pretreatment MRI imaging to re‐evaluate cervical lymph nodal status, including CLN necrosis and infiltration into adjacent structures. MRI diagnosis of lymph node necrosis was based on zones of high signal intensity on T2‐weighted images or zones of low signal intensity on contrast‐enhanced T1‐weighted images.^17^ CLN invasion into adjacent parotid glands, skin, muscles was identified according to the following criteria: (1) the boundary between a cervical lymph node and adjacent tissues was quite vague and disappeared totally, and (2) on MRI, adjacent tissues presented a hypointense signal in the T1‐weighted sequence, hyperintense signal in the T2‐weighted sequence, obvious enhancement in the postcontrast‐enhanced sequence and limited diffusion in the dispersion‐weighted sequence.^18^ After discussion, these rules were followed by both centers.

### 2.4 Statistical analysis

The patients were re‐evaluated every year following the completion of the treatment. OS refers to the period from histological diagnosis to death or last follow‐up. Progression‐free survival (PFS) refers to the period from histological diagnosis to the date of the first failure of treatment, last follow‐up, or death. Distant metastasis‐free survival (DMFS) refers to the period from histological diagnosis to the first distant metastasis or last follow‐up. Locoregional relapse‐free survival (LRFS) refers to the period from histological diagnosis to the first locoregional relapse or the last follow‐up visit. R software (version 4.0.2) and SPSS (version 25) were utilized for all statistical analyses. To examine the differences in clinical characteristics and clinical toxicity, chi‐square or Fisher's exact tests were performed. To minimize the effects of potential confounding factors, we matched the two cohorts at a 1:1 ratio by using propensity score matching (PSM).^19^ A Cox proportional hazards model was applied in both univariate and multivariate analyses, and variables with *p* < 0.1 in the univariate analysis were qualified for inclusion in the multivariate analysis. Hazard ratios (HRs) with 95% confidence intervals (CIs) and independent prognostic variables were determined for OS and PFS with the Cox proportional hazards model. We used the “rms” package to develop a nomogram based on independent predictors associated with PFS. The concordance index (C‐index) and time‐dependent receiver operating characteristic (ROC) curve were employed to evaluate the discrimination ability of the nomograms, and 1000 bootstrap resamples were used to further assess the consistency of the nomograms. These results are shown in calibration curves. OS, PFS, DMFS and LRFS were evaluated using Kaplan–Meier analysis. Tests were all bilateral, and results from the multivariate analysis are expressed in terms of the risk ratio (HR) and the 95% CI. All results were considered statistically significant when *p* < 0.05.

## 3 RESULTS

### 3.1 Clinical characteristics and survival outcomes

1376 LA‐NPC patients who met the eligibility criteria were enrolled in this study, including 560 patients who received AC (range of 1–4 cycles) and 81 patients who received S‐1 (range of 2–24 cycles). Among the 1376 patients, 239 (17.4%) died, 124 (9.0%) developed locoregional recurrence, and 217 (15.8%) developed distant metastasis. The post‐treatment follow‐up period ranged from 2 to147 months, and the median follow‐up duration was 69 months. The 5‐year OS, PFS, DMFS and LRFS rates were 82.4%, 77%, 83.7% and 90.1%, respectively. The detailed clinical characteristics of the 1376 patients are shown in Table 1.

**TABLE 1:** Patient characteristics before and after PSM in the IC+CCRT+AC/MC group and IC+CCRT group

Item	Entire cohort (%)			Propensity score‐matched cohort (%)		
	IC+CCRT+AC/MC	IC+CCRT	*p*	IC+CCR+AC/MC	IC+CCRT	*p*
Total	641 (46.5)	735 (53.5)		396 (50.0)	396 (50.0)	
Age (y)						
<47	356 (55.5)	357 (48.6)	0.010	207 (52.3)	209 (52.8)	0.887
≥47	285 (44.5)	378 (51.4)		189 (47.7)	187 (47.2)	
Sex						
Male	474 (73.9)	535 (72.8)	0.628	293 (74.0)	279 (70.5)	0.267
Female	167 (26.1)	200 (27.2)		103 (26.0)	117 (29.5)	
T stage						
T1	27 (4.2)	40 (5.4)	0.250	18(4.5)	18 (4.5)	0.891
T2	138 (21.5)	143 (19.5)		90 (22.7)	81 (20.5)	
T3	310 (48.4)	384 (52.2)		201 (50.8)	206 (52.0)	
T4	166 (25.9)	168 (22.9)		87 (22.0)	91 (23.0)	
N stage						
N0	21 (3.3)	35 (4.8)	0.357	13 (3.3)	17 (4.3)	0.849
N1	148 (23.1)	159 (21.6)		91 (23.0)	85 (21.5)	
N2	368 (57.4)	407 (55.4)		220 (55.6)	224 (56.6)	
N3	104 (16.2)	134 (18.2)		72 (18.1)	70 (17.6)	
Clinical stage						
III	388 (60.5)	462 (62.9)	0.376	249 (62.4)	249 (62.9)	0.883
IVA	253 (39.5)	273 (37.1)		149 (37.6)	147 (37.1)	
Smoking						
Yes	269 (42.0)	294 (40.0)	0.459	164 (41.4)	150 (37.9)	0.309
No	372 (58.0)	441 (60.0)		232 (58.6)	246 (62.1)	
DDP/NDP dose of CCRT (mg/m^2^)						
<200	496 (77.4)	562 (76.5)	0.687	306 (77.3)	303 (76.5)	0.800
≥200	145 (22.6)	173 (23.5)		90 (22.7)	93 (23.5)	
The number of NDP/DDP in IC stage						
DDP	252 (39.3)	266 (36.2)	0.242	189 (47.7)	163 (41.2)	0.074
NDP	389 (60.7)	469 (63.8)		207 (52.3)	233 (58.8)	
The number of NDP/DDP in CCRT stage						
DDP	211 (32.9)	277 (32.1)	0.071	156 (39.4)	143 (36.1)	0.379
NDP	430 (67.1)	458 (67.9)		240 (60.6)	253 (63.9)	
IC regimen						
TPF	149 (23.2)	197 (26.8)	0.129	93 (23.5)	74 (18.7)	0.098
TP/PF	492 (76.8)	538 (73.2)		303 (76.5)	322 (81.3)	
Number of IC cycles						
<2	178 (27.8)	178 (24.2)	0.133	95 (24.0)	92 (23.2)	0.802
≥2	463 (72.2)	557 (75.8)		301 (76.0)	304 (76.8)	
BMI (kg/m^2^)						
<22	287 (44.8)	353 (48.0)	0.227	177 (44.7)	185 (46.7)	0.568
≥22	354 (55.2)	382 (52.0)		219 (55.3)	211 (53.3)	
Histology						
I/II	19 (3.0)	19 (2.6)	0.669	12 (3.0)	12 (3.0)	1.000
III	622 (97.0)	716 (97.4)		384 (97.0)	384 (97.0)	
CLN necrosis						
Yes	200 (31.2)	229 (31.2)	0.986	135 (34.1)	120 (30.3)	0.254
No	441 (68.8)	506 (68.8)		261 (65.9)	276 (69.7)	
CLN infiltration into adjacent structures						
Yes	59 (9.2)	55 (7.5)	0.248	41 (10.4)	35 (8.8)	0.469
No	582 (90.8)	680 (92.5)		355 (89.6)	361 (91.2)	

Abbreviations: BMI, body mass index; CCRT, concurrent chemoradiotherapy; CLN, cervical lymph node; DDP/NDP, cisplatin/nedaplatin; IC, induction chemotherapy; PF, cisplatin/nedaplatin and fluorouracil; TP, cisplatin/nedaplatin and docetaxel/paclitaxel; TPF, cisplatin/nedaplatin, docetaxel/paclitaxel and fluorouracil.

**FIGURE 1:**
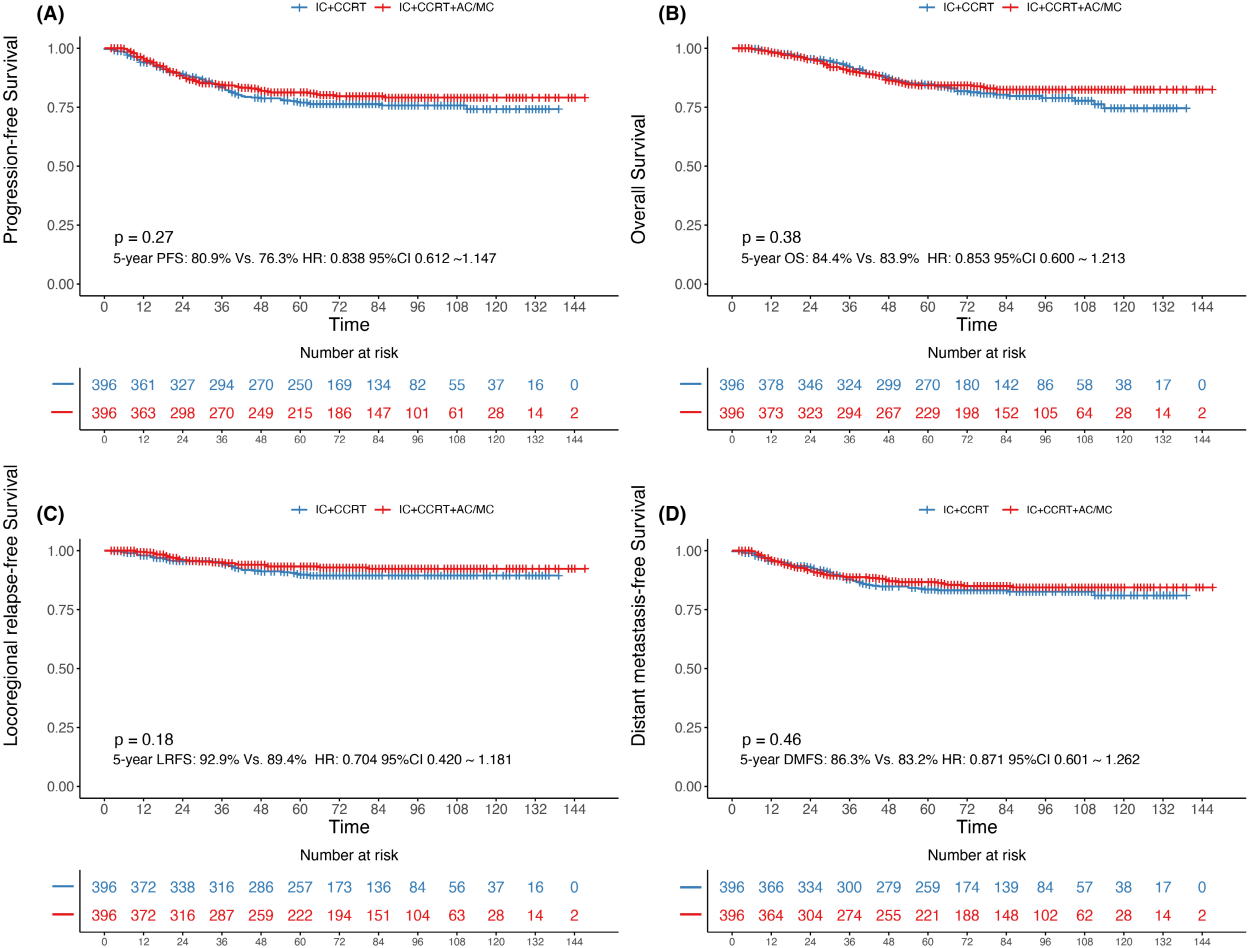
Kaplan–Meier analysis of overall survival (OS), progression‐free survival (PFS), distant metastasis‐free survival (DMFS) and locoregional relapse‐free survival (LRFS) between the IC+CCRT and IC+CCRT+AC/MC groups after PSM.

Following PSM, all of the clinical characteristics were balanced between IC+CCRT+AC/MC group and IC+CCRT group (all *p* > 0.05), and every group consisted of 396 patients (Table 1). The median follow‐up duration was 70 months (range of 2–147 months). The 5‐year OS, PFS, DMFS and LRFS were 84.6%, 78.5%, 84.3% and 91.3%, respectively. As shown in Figure 1, the 5‐year OS, PFS, DMFS and LRFS rates of these two cohorts were 84.9% versus 83.9% (*p* = 0.38), 80.9% versus 76.3% (*p* = 0.27), 86.3% versus 83.2% (*p* = 0.46) and 92.9% versus 89.4% (*p* = 0.18), respectively. Therefore, the IC+CCRT+AC/MC treatment protocol did not prolong survival in patients with LA‐NPC.

### 3.2 Establishment of a nomogram for OS and PFS

Following univariate and multivariate analyses, all variables with statistical significance (*p* < 0.05) or obvious clinical relevance were added to the regression analysis model. Sex, N stage, BMI, CLN necrosis and CLN infiltration into adjacent structures were considered independent factors for OS (Table 2); N stage, DDP/NDP dose of CCRT, IC cycles, IC regimen, BMI, CLN necrosis and CLN infiltration into adjacent structures were considered independent factors for PFS (Table 2).

**TABLE 2:** Univariate and multivariate analyses of OS and PFS in the 1376 LA‐NPC patients

Variable	Univariate analysis			*p*	Multivariate analysis			*p*
	OS	*p*	PFS		OS	*p*	PFS	
	HR (95% CI)		HR (95% CI)		HR (95% CI)		HR (95% CI)	
Sex (male vs. female)	0.698 (0.511–0.953)	0.024	0.779 (0.597–1.018)	0.067	0.730 (0.532–1.002)	0.051	0.788 (0.601–1.032)	0.187
Age (<46 vs. ≥ 46)	1.265 (0.981–1.631)	0.070	1.148 (0.917–1.437)	0.227	1.189 (0.919–1.538)	0.239	—	
Smoking (yes vs. no)	1.210 (0.938–1.561)	0.143	1.220 (0.973–1.529)	0.084	—		1.036 (0.800–1.342)	0.672
T stage	1.097 (0.933–1.290)	0.262	1.086 (0.941–1.253)	0.261	—		—	
N stage	1.608 (1.336–1.937)	<0.001	1.692 (1.433–1.998)	<0.001	1.218 (1.040–1.485)	0.019	1.338 (1.120–1.598)	<0.001
Clinical stage (III vs. IV)	2.312 (1.789–2.987)	<0.001	2.210 (1.763–2.770)	<0.001	Not selected		Not selected	
DDP/NDP dose of CCRT (<200 mg/m^2^ vs. ≥200 mg/m^2^)	0.877 (0.646–1.190)	0.399	0.778 (0.588–1.031)	0.080	—		0.761 (0.561–1.030)	0.080
The number of NDP/DDP in CCRT stage	1.670 (0.651–3.730)	0.330	1.150 (0.661–1.860)	0.722	—		—	
The number of NDP/DDP in CCRT stage	1.270 (0.701–2.35)	0.456	1.456 (0.978–1.871)	0.096	—		—	
IC regimen (TPF vs. TP/PF)	0.794 (0.579–1.063)	0.122	0.771 (0.600–0.990)	0.042	—		0.808 (0.623–1.048)	0.086
IC cycles	0.759 (0.456–0.996)	0.047	0.749 (0.588–0.954)	0.019	0.848 (0.644–1.117)	0.199	0.784 (0.614–1.002)	0.025
BMI (<22 kg/m^2^vs. ≥22 kg/m^2^)	0.695 (0.539–0.897)	0.005	0.741 (0.592–0.928)	0.009	0.653 (0.504–0.845)	<0.001	0.721 (0.574–0.906)	0.002
Histology (I/II vs. III)	0.760 (0.390–1.479)	0.419	0.848 (0.452–1.593)	0.608	—		—	
CLN necrosis (yes vs. no)	2.348 (1.822–3.025)	<0.001	2.109 (1.684–2.642)	<0.001	1.696 (1.292–2.227)	<0.001	1.672 (1.315–2.124)	<0.001
CLN infiltration into adjacent structures (yes vs. no)	3.825 (2.858–5.119)	<0.001	2.994 (2.272–3.946)	<0.001	2.141 (1.512–3.030)	<0.001	1.703 (1.238–2.343)	<0.001

Abbreviations: BMI, body mass index; CCRT, concurrent chemoradiotherapy; CLN, cervical lymph node; DDP/NDP, cisplatin/nedaplatin; IC, induction chemotherapy; PF, cisplatin/nedaplatin and fluorouracil; TP, cisplatin/nedaplatin and docetaxel/paclitaxel; TPF, cisplatin/nedaplatin, docetaxel/paclitaxel and fluorouracil.

**FIGURE 2:**
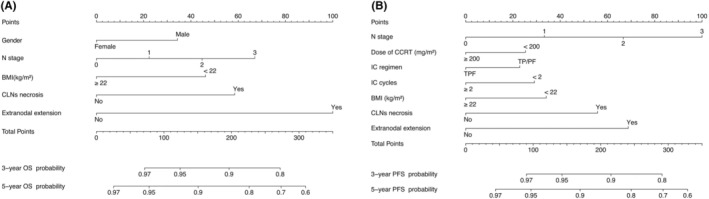
(A) Nomograms to predict overall survival (OS); (B) Nomograms to predict progression‐free survival (PFS). Each variable corresponds to a score on the point scale. After adding the total points, the predicted survival possibility is determined by projecting the total points to the survival axis.

**FIGURE 3:**
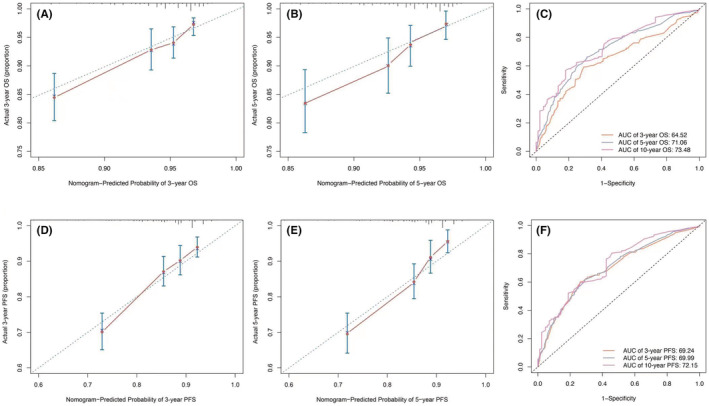
Calibration curves of the nomogram‐predicted probability of 3‐ and 5‐year OS (A) and (B); PFS (D) and (E). The AUCs of 5‐years OS (C) and PFS (F). The td‐receiver operator characteristic curves of the nomogram of OS and PFS; OS: overall survival; PFS: progression‐free survival.

Based on the selection of independent prognostic factors, we developed 3‐ and 5‐year OS and PFS nomograms (Figure 2A,B).^20^ The Harrell C‐index values for PFS and OS predictions were 0.667 (95% CI 0.636–0.699) and 0.682 (95% CI 0.647–0.718), respectively. Calibration curves were used to demonstrate good consistency between actual observations of 3‐ and 5‐year OS and PFS and the nomogram prediction (Figure 3A,B and Figure 3D,E).^21^

Time‐dependent ROC curves illustrated the good discrimination ability of our nomograms, and the AUCs of 5‐year OS and PFS were 71.05% and 69.99%, respectively, as shown in Figure 3C,F.

### 3.3 Risk stratification for PFS

**FIGURE 4:**
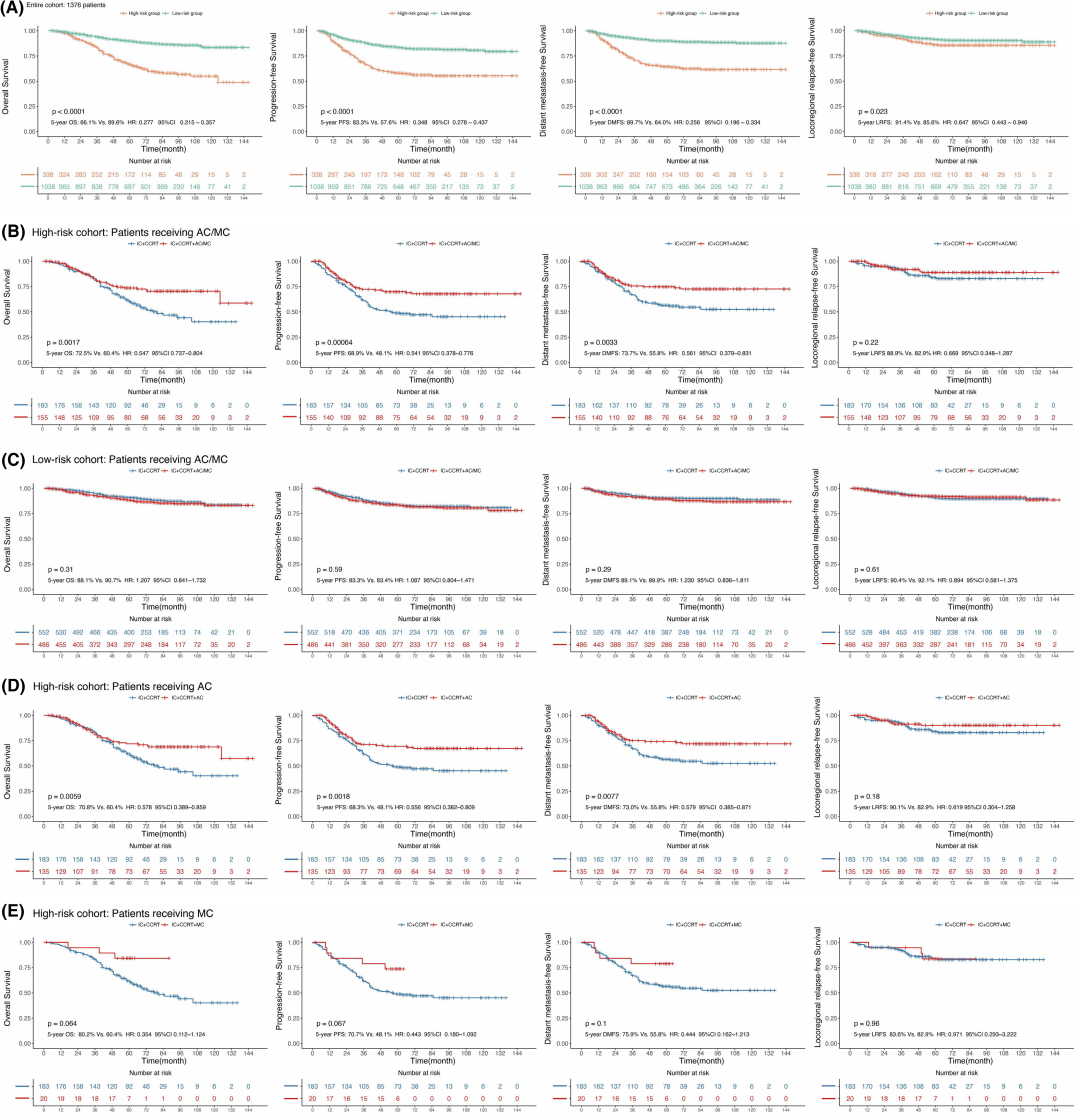
(A) Kaplan–Meier curves are shown for overall survival (OS), progression‐free survival (PFS), distant metastasis‐free survival (DMFS), locoregional relapse‐free survival (LRFS) between the high‐risk cohort (total score ≥160) and the low‐risk cohort (total score <160); (B) Kaplan–Meier analysis of OS, PFS, DMFS and LRFS between IC+CCRT and IC+CCRT+AC/MC in the high‐risk cohort; (C) Kaplan–Meier analysis of OS, PFS, DMFS and LRFS between IC+CCRT and IC+CCRT+AC/MC in the low‐risk cohort; (D) Kaplan–Meier analysis of OS, PFS, DMFS and LRFS between IC+CCRT and IC+CCRT+AC in the high‐risk cohort; and (E) Kaplan–Meier analysis of LRFS, PFS, DMFS and OS between IC+CCRT and IC+CCRT+MC in the high‐risk cohort.

Based on the nomogram, a total score was calculated, and 338 patients with a total score ≥160 were assigned to the high‐risk group; 1038 patients with a total score <160 were assigned to the low‐risk group. The low‐risk group had better 5‐year OS, PFS, DMFS and LRFS (all *p* < 0.01) than the high‐risk group (Figure 4A).

### 3.4 Clinical value of AC and/or MC in the high‐risk group

There were 338 patients in the high‐risk group: 155 (45.9%) patients received IC+CCRT+AC/MC, and 183 (54.1%) patients received IC+CCRT. The median follow‐up duration was 62 months (range of 2–147 months). The 5‐year OS, PFS, DMFS and LRFS rates in the high‐risk group were 66.3%, 56.5%, 63.5% and 84.5%, respectively.

The 5‐year LRFS, PFS, DMFS and OS rates in the IC+CCRT+AC/MC and IC+CCRT groups were 72.5% vs. 60.4% (*p* = 0.0017), 68.9% vs. 48.1% (*p* = 0.00064), 73.7% vs. 55.8% (*p* = 0.0033), and 88.9% vs. 82.9% (*p* = 0.22), respectively (Figure 4B) in the high‐risk group. There was no difference in the low‐risk group between the IC+CCRT+AC/MC and IC+CCRT cohorts (Figure 4C). The clinical values of AC and MC were analyzed separately. Compared with IC+CCRT, the addition of AC could significantly improve the 5‐year OS (70.8% vs. 60.4%; *p* = 0.0059), PFS (68.3% vs. 48.1%; *p* = 0.0018) and DMFS (73.0% vs. 55.8% *p* = 0.0077) rates (Figure 4D). However, the addition of MC only improved the 5‐year OS (80.2% vs. 60.4%; *p* = 0.064) rate compared with IC+CCRT, but the PFS (70.7% vs. 48.1%; *p* = 0.067), DMFS (75.9% vs. 55.8%; *p* = 0.10) and LRFS (83.6% vs. 82.9%; *p* = 0.96) rates were not significantly improved (Figure 4E).

### 3.5 Adverse events

The IC+CCRT+AC cohort showed a much higher incidence of grade 3/4 leukocytopenia and neutropenia (40.4% and 32.1%, respectively) than the IC+CCRT cohort (24.5% and 25.2%, respectively) (*p* < 0.05). However, hematologic and nonhematological toxicity were similar between the IC+CCRT cohort and the IC+CCRT+MC cohort (all *p* > 0.05), and patients who received S‐1 were more likely to develop skin hyperpigmentation than those receiving other treatment protocols (Table 3).

**TABLE 3:** Adverse events

Adverse event	IC+CCRT regimen (case%)		IC+CCRT+AC regimen (case%)		*p* value	IC+CCRT regimen (case%)		IC+CCRT+MC regimen (case%)		*p* value
Hematological										
	Grade 0–2	Grade 3–4	Grade 0–2	Grade 3–4		Grade 0–2	Grade 3–4	Grade 0–2	Grade 3–4	
Leukocytopenia	555 (75.5)	180 (24.5)	334 (59.6)	226 (40.4)	<0.001	555 (75.5)	180 (24.5)	57 (70.4)	24 (29.6)	0.311
Neutropenia	550 (74.8)	185 (25.2)	380 (67.9)	180 (32.1)	0.006	550 (74.8)	185 (25.2)	63 (77.8)	18 (22.2)	0.560
Anemia	687 (93.5)	48 (6.5)	519 (92.7)	41 (7.3)	0.577	687 (93.5)	48 (6.5)	73 (90.1)	8 (9.9)	0.258
Thrombocytopenia	708 (96.3)	27 (3.7)	538 (96.1)	22 (3.9)	0.812	708 (96.3)	27 (3.7)	78 (96.3)	3 (3.7)	1.000
Nonhematological										
Liver function	720 (98.0)	15 (2.0)	554 (98.9)	6 (1.1)	0.171	720 (98.0)	15 (2.0)	81 (100)	0 (0)	0.385
Nausea/vomiting	685 (93.3)	49 (6.7)	533 (95.2)	27 (4.8)	0.160	685 (93.3)	49 (6.7)	75 (92.6)	6 (7.4)	0.803
Oral mucositis	571 (77.7)	164 (22.3)	449 (80.2)	111 (19.8)	0.277	571 (77.7)	164 (22.3)	61 (75.3)	20 (24.7)	0.627

	IC+CCRT regimen (case%)		IC+CCRT+AC regimen (case%)			IC+CCRT regimen (case%)		IC+CCRT+MC regimen (case%)		
	Grade 0	Grade 1–2	Grade 0	Grade 1–2		Grade 0	Grade 1–2	Grade 0	Grade 1–2	
Skin hyperpigmentation	735 (100)	0 (0)	560 (0)	0 (0)	1.000	735 (0)	0 (0)	66 (81.5)	15 (18.5)	<0.001
Renal function	640 (87.1)	95 (12.9)	466 (83.2)	94 (16.8)	0.051	640 (87.1)	95 (12.9)	74 (91.4)	7 (8.6)	0.269

Abbreviations: AC, adjuvant chemotherapy; CCRT, concurrent chemoradiotherapy; IC, induction chemotherapy; MC, maintenance chemotherapy.

## 4 DISCUSSION

Based on our knowledge, our study is the first to combine clinical data and radiographic data to identify indications for AC/MC after IC+CCRT based on a large retrospective sample. In this study, a prognostic nomogram was established that included N stage, DDP/NDP dose of CCRT, IC cycles, IC regimen, BMI and CLN necrosis and CLN infiltration into adjacent tissues, and then, the prognostic nomogram was used to divide 1376 LA‐PNC patients into high‐risk and low‐risk cohorts. Patients with high‐risk NPC treated with the IC+CCRT+AC/MC regime showed enhanced 5‐year OS, PFS and DMFS rates; the same efficacy was observed in patients with low‐risk NPC who received IC+CCRT+AC/MC or IC+CCRT. Therefore, we believe that IC+CCRT+AC/MC is an effective therapeutic strategy for patients with high‐risk NPC, further reducing the incidence of distant metastasis.

After the Intergroup 0099 trial, CCRT plus AC with PF became the optimum treatment strategy for LA‐NPC patients^22^; since then, the CCRT scheme has been validated by a large number of prospective studies and widely applied for II–IVa NPC.^23^, ^24^, ^25^ A phase III clinical trial conducted in 2012 showed that CCRT plus AC using PF and CCRT alone has similar survival outcomes due to poor tolerance after CCRT, and this conclusion makes the use of AC after CCRT controversial.^26^, ^27^ Subsequently, Sun et al. proved that adding IC using TPF to CCRT could prolong OS and result in a lower risk of distant metastasis in LA‐NPC^5^; thus, IC+CCRT has become a standard therapy (category 2A) that is recommended by NCCN guidelines.^4^ However, according to a pooled analysis of individual patient data (IPD), although complete clinical remission was achieved in the vast majority of patients after treatment with IC+CCRT, approximately 30% of LA‐NPC patients experienced failure treatment including local, regional recurrence and/or distant metastasis.^6^ Therefore, the intensity of LA‐NPC treatment based on IC+CCRT may need further enhancement, and the value of adjuvant chemotherapy after IC+CCRT is worthy of exploration.

In fact, evidence suggests that AC can eliminate the potential residual cancer cells in the local area and subclinical metastases and delay the occurrence of distant metastasis in solid tumors.^28^, ^29^ In Chen et al.'s study, 40% of patients failed to complete 3 cycles of AC as scheduled, which included 44 patients who did not receive the complete regimen of AC because of poor tolerance after CCRT; this is the primary cause of failure in this study.^26^, ^27^ In a pooled analysis of IPD from 20 clinical trials, it was determined that adding AC to CCRT was the best therapy option for LA‐NPC than other regimens in term of distant control (DC), OS and PFS rates.^30^ According to the latest version of the NCCN, though the adjuvant chemotherapy (AC) with capecitabine is category 2B according to the latest version of the NCCN guideline, Majun et al revealed that LA‐NPC patients treated with capecitabine achieved a higher 3 year‐OS (*p* < 0.05).^4^, ^9^ Therefore, whether the addition of AC or MC to IC+CCRT could further prolong the survival is worth further exploration.

Cisplatin plus docetaxel (TP) is commonly used as a curative option for IC in clinical practice. Docetaxel is a microtubule‐stabilizing drug.^31^ TAX 323/324 studies have shown that adding docetaxel to PF (TPF) could further enhance survival rates in head and neck cancers.^32^, ^33^ Moreover, according to a large‐scale retrospective analysis, the TPF regimen is more effective than the PF regimen among patients with LA‐NPC.^34^ Therefore, docetaxel has clinical efficacy in treating LA‐NPC. Additionally, Zhang et al. and Tao et al. showed that AC using TP was an effective treatment option for LA‐NPC patients, and most patients tolerated it.^10^, ^35^ Cisplatin plus 5‐fluorouracil (PF) is the only AC treatment option recommended by the NCCN guidelines to date.^4^ 5‐Fluorouracil can effectively damage tumor cells since it can inhibit thymidine synthase and affect DNA synthesis and is widely used in a variety of solid tumors.^36^ The clinical value of the PF regimen has been repeatedly demonstrated and widely accepted when it is applied as an AC or IC.^22^, ^34^ Capecitabine and S‐1 are both orally administered anticancer drugs that can be transformed into 5‐fluorouracil in the body. Recently, an increasing number of studies have indicated that S‐1 has better clinical effects for patients with LA‐NPC. In a prospective study, N3‐positive patients who received S‐1 for a long time had mild side effects and tolerated the treatment well.^37^ Hence, MC with S‐1 is a reasonable option. However, we used PSM to balance the two cohorts of patients who underwent the IC+CCRT or IC+CCRT+AC/MC therapeutic protocol, and the results showed that two cohorts have similar survival outcomes. This result is consistent with Zou et al.'s and Tao et al.'s study.^10^, ^38^ Chen et al. performed a study to explore the correlation between postradiotherapy (RT) EB‐DNA levels and AC with cisplatin plus gemcitabine, and they found that AC did not benefit patients who had high levels of EB‐DNA after RT.^39^ These research conclusions indicate that we may need to further investigate the clinical value of AC from other perspectives.

Numerous studies have shown that pretreatment CLN status is closely related to the incidence of distant metastasis and has been considered an indicator of poor prognosis. A retrospective study showed that patients with cervical lymph infiltration into adjacent structures may have a higher probability of distant metastasis after treatment completion.^12^, ^13^ Another study indicated that patients with CLN necrosis had worse survival outcomes than those without CLN necrosis.^11^ After univariate and multivariate analyses, our study found a similar result as in previous studies.

In fact, IC cycles, IC regimens and cumulative DDP doses during radiotherapy have always been a research hotspot. Three cycles of IC have commonly been used in many prospective cohort studies. Nevertheless, most retrospective analyses have found that two and three cycles have the same effects.^40^, ^41^, ^42^ Our research showed that patients who received one cycle of IC had worse survival outcomes than those who received two or more cycles. Recently, two clinical retrospective studies found that the TPF regimen led to better 5‐PFS and DMFS survival in LA‐NPC patients than the PF or TP regimen, which was similar to our finding.^34^, ^43^ In addition to IC cycles and IC regimens, many studies have shown that a cumulative dose of 200 mg/m^2^ cisplatin is optimal for treating patients with LA‐NPC.^44^, ^45^, ^46^ However, the optimal cumulative dose of cisplatin during radiotherapy after IC remains controversial, so we still regarded 200 mg/m^2^ as the critical dose. Numerous studies have demonstrated that BMI is an independent prognostic factor for solid tumors. Wang et al.'s study showed that low BMI before treatment is an indicator for poor prognosis in LA‐NPC patients, similar to our finding.^47^

These prognosis‐associated indicators were adopted to establish a nomogram for OS and PFS. Patients were then divided into low‐ and high‐risk groups based on the nomogram. Low‐risk patients had a lower disease progression rate and a better OS than high‐risk patients. Based on this result, we found that insufficient treatment intensity of IC+CCRT and/or CLN status can increase the risk of disease progression after treatment. This result indicates that the IC+CCRT+AC/MC treatment option might significantly improve 5‐year overall survival by reducing the risk of distant metastasis in the high‐risk group; at the same time, we found that the treatment intensity of IC+CCRT was sufficient in patients with low‐risk NPC. Thus, adding AC/MC to IC+CCRT is not only a remedy for undertreated patients but also a better curative option for patients with CLN necrosis and/or infiltration into adjacent structures. In subgroup analysis, IC+CCRT+AC further improved OS by reducing the odds of DMFS (all *p* < 0.05). Nevertheless, this study failed to demonstrate clinical efficacy of S‐1 because high‐risk patients did not benefit from the IC+CCRT+MC therapeutic protocol. However, Zhu et al.'s study showed that MC using S‐1 or capecitabine could further improve OS in high‐risk patients.^48^ We presume that the main reason for the negative result in this study is that only twenty high‐risk patients received IC+CCRT+MC by comparison with Zhu et al.'s study; thus, the relatively small sample size was not sufficient for a statistically positive result, despite our data displaying a trend toward statistical significance between the IC+CCRT+MC cohort and the IC+CCRT cohort.

The IC+CCRT+AC cohort had a higher grade 3/4 leukocytopenia/neutropenia toxicity than the IC+CCRT cohort, which was similar to Zou et al.'s study.^38^ Compared to the standard treatment regimen, the IC+CCRT+MC treatment regimen did not increase the incidence of nonhematological and hematological adverse events, which is consistent with previous studies. S‐1 was associated with hand‐foot syndrome, and most patients could tolerate it.

There are some limitations in this study. First, although PSM was used to balance the two cohorts, some biases remained in this retrospective study. Second, because most patients do not have EB‐DNA records, we could not correlate plasma EB‐DNA levels with CLN status and clinical data. Third, compared to histology, some mild CLN necrosis was rarely identified by MRI, but CLN infiltration into adjacent structures is more easily detected by MRI. Therefore, in the future, our studies should be further confirmed by a prospective clinical study.

## 5 CONCLUSION

This study re‐evaluated the clinical value of AC in the context of the popularity of the IC+CCRT treatment regimen by analyzing clinical data and CLN status. A prognostic nomogram was developed that included N stage, DDP/NDP dose of CCRT, IC cycles, IC regimen, BMI, CLN necrosis and CLN infiltration into adjacent structures. This study found that AC/MC could decrease the occurrence of distant metastasis and further prolong OS in the high‐risk group. The incidence of toxic effects of IC+CCRT+AC was significantly increased compared with that of IC+CCRT, especially the incidence of leukocytopenia and neutropenia. However, IC+CCRT and IC+CCRT+MC were associated with similar incidences of toxic effects. Our study suggests that the addition of AC/MC to IC+CCRT can notably increase the therapeutic effects in high‐risk patients with LA‐NPC and is valuable in clinical practice.

## AUTHOR CONTRIBUTIONS

**Hao‐Yun Tao:** Conceptualization (equal); data curation (equal); formal analysis (equal); writing – original draft (equal); writing – review and editing (equal). **Fang He:** Data curation (equal); writing – original draft (equal); writing – review and editing (equal). **Qiyun Shi:** Formal analysis (equal). **Ran Liu:** Data curation (equal). **Hui Liu:** Data curation (equal).

## CONFLICT OF INTEREST

The authors declare no conflicts of interest.

## Data Availability

Data sharing is not applicable to this article as no new data were created or analyzed in this study.
